# Gender incongruence: A longitudinal perspective from childhood throughout adolescence

**DOI:** 10.1192/j.eurpsy.2021.2159

**Published:** 2021-08-13

**Authors:** L. Borges, G. Riggi

**Affiliations:** 1 Psiquiatria, CHUA - Unidade de Portimão, Portimão, Portugal; 2 Serviço De Psiquiatria E Saúde Mental Da Infância E Da Adolescência, Hospital de São Francisco Xavier, Lisboa, Portugal

**Keywords:** childhood, adolescence, gender incongruence, gender dysphoria

## Abstract

**Introduction:**

Gender identity is each person’s internal and individual experience of gender. Gender expression is how a person publicly expresses their gender. Gender incongruence (GI) is defined as a condition in which a person has a marked incongruence between the expressed or experienced gender and the biological sex at birth. Adolescence is a crucial period for the persistence or development of GI, due hormonal changes, peer relations and first romantic experiences.

**Objectives:**

To make a revision of the literature about GI along childhood throughout adolescence.

**Methods:**

Research in the literature with the words “gender identity”, “gender incongruence”, “gender dysphoria”, “childhood” and “adolescence” in scientific databases.

**Results:**

GI is present in a small percentage of children, often provoking psychopathological distress. There is a high prevalence of autism spectrum disorders in these children, compared with the general population. In most cases the dysphoria does not persist until adolescence. There has been an increasing number of adolescents seeking for treatment at gender identity services. The studies show that after the onset of puberty, the probability of persistent GI is high and that adolescents submitted to hormonal suppression tend to continue the medical treatment.

**Conclusions:**

Epidemiological formal studies about gender incongruence in children and adolescents are very few. Studies of prevalence in these populations are community studies and don’t reflect the true prevalence of GI, so it would be necessary to investigate its prevalence and persistence in different world populations. It’s also necessary to make more prospective studies about the long-term effects of the medical treatment of GI.

**Disclosure:**

No significant relationships.

